# Evaluating the efficiency of two different over-the-counter tooth whitening systems: a randomised controlled clinical trial

**DOI:** 10.1038/s41405-024-00227-2

**Published:** 2024-05-31

**Authors:** Eenass Krayem, Avijit Banerjee, Hussam Milly

**Affiliations:** 1 Faculty of Dental Medicine, Damascus University, Damascus, Syria; 2 Centre of Oral Clinical Translational Sciences/Dept. of Conservative & MI Dentistry, Faculty of Dentistry, Oral & Craniofacial Sciences, King’s College London, Guy’s Dental Hospital, London, SE1 9RT UK

**Keywords:** Tooth whitening, Dental materials

## Abstract

**Objectives:**

To compare whitening efficiency and tooth sensitivity (TS) of two different over the counter (OTC) whitening systems in comparison to conventional at-home bleaching using 20% carbamide peroxide.

**Materials and methods:**

A randomised controlled clinical trial was conducted with three parallel groups (*n* = 13): (A) at-home whitening using 20% carbamide peroxide (20% CP), (B) OTC ready-to-use gel trays and (C) OTC-paint on gel. Clinical colour change values (ΔE) were measured using spectrophotometry at T0: baseline, T1: 7-day and T2: 14-day from whitening start, T3: 2 weeks and T4: 6 months after whitening end. TS was recorded using a visual analogue scale (VAS). ΔE and TS values were statistically analysed. The level of significance for all tests was 5%.

**Results:**

Significant differences in ∆E values were recorded between the experimental groups. ∆E values were significantly higher in the 20% CP conventional at-home whitening group. TS measurements were significantly higher in the 20% CP whitening group (*p* < 0.05).

**Conclusion:**

Conventional at-home whitening revealed significantly improved colour change when compared to the OTC-paint on gel and OTC ready-to-use gel tray whitening systems. There was a significant colour relapse in OTC systems.

**Clinical relevance:**

The use of tested OTC systems is not recommended as they are not effective in a clear and prolonged improved shade change. Conventional at-home whitening using 20% CP showed higher whitening efficiency and colour stability. This trial was registered with a International Standard Randomised Controlled Trial Number (ISRCTN23096480), Registration date: 12/04/2023.

## Introduction

Tooth discoloration is one of the most common reasons to seek dental aesthetic procedures [1]. Tooth whitening (bleaching) is an effective, non-invasive therapeutic method to manage tooth discoloration [2]. Various whitening protocols are available, including dentist-supervised-at-home and in-office procedures which utilise different concentrations of carbamide peroxide (CP) and hydrogen peroxide (HP) [3]. Using an oxidation/reduction reaction, hydrogen peroxide breaks down chromogenic molecules in dentine and enamel, resulting in the conversion of pigment ring structures into terminal carboxylic acids. As a result, the tooth’s colour is lightened and the acids are expelled from its surface and structure [4–6].

Over-the-counter (OTC) whitening products have been introduced as an alternative approach to dentist-supervised whitening, with a lower cost and ease of availability. OTC whitening products sector have played a significant role in the growth of the global tooth whitening market which is anticipated to reach over $2 billion in 2024 [7, 8]. The ‘DIY ‘nature of OTC products, however, arouses concerns about the risk of misuse, overuse and/or abuse [9, 10]. OTC products are available in shops, pharmacies and online stores and customers can obtain and use them easily without any medical / dental diagnosis or supervision [11, 12]. This approach ignores important clinical interventions including a thorough dental examination, diagnosis and determination of the aetiology of the discoloration, caries susceptibility / management, replacing failed restorations and hypersensitivity management [13].

Different OTC whitening products and types are available in the market including strips, rinses, dentifrices, paint-on films and prefabricated trays including gel [14, 15]. The efficiency/safety evaluation of OTC whitening products revealed conflicting outcomes. The variances observed in previous studies can be attributed to the utilisation of distinct analytical methods and OTC types [16]. In addition, a limited number of carefully planned clinical trials regarding their evaluation have been reported in the dental literature. Therefore, this randomised controlled clinical trial explored the whitening efficiency and tooth sensitivity associated with two popular OTC systems in the Middle East (OTC-paint on gel and OTC-ready to use trays with gel) in comparison with a conventional at-home dental whitening protocol with 20% carbamide peroxide. The tested OTC systems in the present study are available at a convenient price for the majority of people in the Middle East. The null hypothesis investigated was that there were no differences in the efficiency and tooth sensitivity between the three tested whitening systems after a 6-month clinical follow-up.

## Materials and methods

### Ethics approval and protocol registration

This randomised controlled clinical trial was approved and registered with an International Standard Randomised Controlled Trial Number (ISRCTN23096480), Registration date: 12/04/2023. This study follows the Consolidated Standards of Reporting Trials (CONSORT) statement [17], and was conducted in accordance with the Declaration of Helsinki and was approved by the Damascus University Research Ethics Committee (ref: MS3833). This trial was conducted in the Faculty of Dental Medicine, Damascus University.

### Sample size and recruitment

The sample size was calculated using GPower 3.1 (Franz Faul, Kiel University, Kiel, Germany), using the t-student statistical test for two independent samples with a statistical power of 95% and a significance level 5%. In accordance with a previous study, it was necessary to recruit 10 participants per group to detect a significance of a clinical difference of at least a 2.5 increase, assuming a standard deviation of 1.2 [18]. Thirteen participants per group were enroled, taking into consideration the potential loss to follow-up of 62%, with an overall sample size *n* = 39. The inclusion and exclusion criteria are presented in Table 1. All participants in this trial signed an informed consent after a cooling off period.

**Table 1:** Inclusion and exclusion criteria.

*Inclusion criteria*	*Exclusion criteria*
Age: 18 to 35 y	Previous use of whitening agents
Good general/oral health	Orthodontic treatment
No caries/restorations on the six maxillary anterior teeth	Parafunctional habits
Presenting tooth shade: A_2_ or darker	Pregnant/lactating women
No history of tooth sensitivity	Cigarette smoking

### Randomisation and sample allocation

Thirty-nine participants meeting the inclusion criteria were assigned randomly into three experimental groups (*n* = 13); CP: conventional at-home whitening using 20% CP (Opalescence, Ultradent, South Jordan, USA), OTC-WL: WhiteLight^TM^ tooth whitening set and OTC-DW: Dazzling white (paint on gel) system (Table 2). The randomisation was conducted by a staff member, who was not involved in this study, by choosing random allocation of the group codes.

**Table 2:** The manufacturer’s tooth whitening protocols.

*Group Code*	*Tooth Whitening Agents/Manufacturers*	*Composition*	*Application regimen*
CP	At-home bleaching (Opalescence, Ultradent, USA)	20% CP, Glycerine, Water, Xylitol, Sodium Hydroxide, EDTA, Potassium Nitrate, Sodium Fluoride	14 days application, Once daily for 4 h
OTC-WL	OTC; ready to use trays with gel (WhiteLight^TM^, OME, USA)	CP, Water, Glyserin, Povidone, Silica, Sodium Hydroxide, Sodium Saccharin, Sorbitol, EDTA	14 days application, Once daily for 30 min with light activation
OTC-DW	OTC; paint on gel (Dazzling white, DR.Fresh Inc., Canada)	HP, purified water, denatured alcohol, polyvinyl, pyrrolidone, polyethelene, glycol	14 days application, Twice daily for 10 min

### Intervention

A custom tray was fabricated for each patient using soft vinyl sheets, 0.8 mm (Sof-Tray Classic, Ultradent, South Jordan, UT, USA) and trimmed 1 mm beyond the gingival margin. This tray included a hole in the middle of the buccal surface of the left maxillary central incisor to standardise colour change measurement during the follow-up periods. Another custom tray, spaced around the labial surfaces of the anterior teeth, was made for each patient in the at-home whitening group for whitening procedure. Each patient was provided with oral hygiene instruction with a toothbrush and a non-whitening dentifrice (Colgate Total Colgate-Palmolive Company, New York, USA) in order to standardise the daily oral hygiene protocol for all subjects. The whitening products were applied according to the manufacturer’s recommendations. Table 2 presents the experimental groups and the manufacturer regime of the whitening protocols.

### Colour change evaluation

The colour of the teeth was assessed using International Commission on Illumination (CIE) L*, a*, b* parameters. An Easy Shade Advance 4.0 spectrophotometer (VITA Zahnfabrik, Bad Säkingen, Germany) was used on the middle third of the buccal surface of the maxillary right canine and maxillary left central incisor through the holes in the guide tray. Colour measurement was performed at; T0: baseline, T1: 7-day and T2: 14-day from whitening start, T3: 2 weeks and T4: 6 months after whitening end.

The total colour difference ∆E* was calculated by the following equation:

$$\Delta {{{{{\rm{E}}}}}}* ={[{(\Delta {{{{{\rm{a}}}}}}* )}^{2}+{(\Delta {{{{{\rm{b}}}}}}* )}^{2}+{(\Delta {{{{{\rm{L}}}}}}* )}^{2}]}^{1/2}$$

### Tooth sensitivity evaluation

Patients were asked to evaluate tooth sensitivity at (1-day, 2-day, 3-day, 4-day, 5-day, 7-day and 14-day) of whitening using a visual analogue scale (VAS). Patients were asked to draw a vertical line cutting a horizontal line (VAS chart) if they experienced any pain or discomfort in their six maxillary interior teeth. The scores for the sensitivity levels were: 0 = no sensitivity; 1–3 = mild sensitivity; 4–6 = moderate sensitivity, 7–8 = severe sensitivity; 9–10 = unbearable sensitivity.

### Statistical analysis

Statistical analysis was accomplished using SPSS software (v22; SPSS Inc. Chicago, IL, USA). The normal distribution of the data was evaluated using the Kolmogorov- distribution test. Differences in ∆E and tooth sensitivity measurements were analysed using one-way ANOVA and Kruskal Wallis tests respectively. The level of significance for all tests was 5%.

## Results

**Fig. 1:**
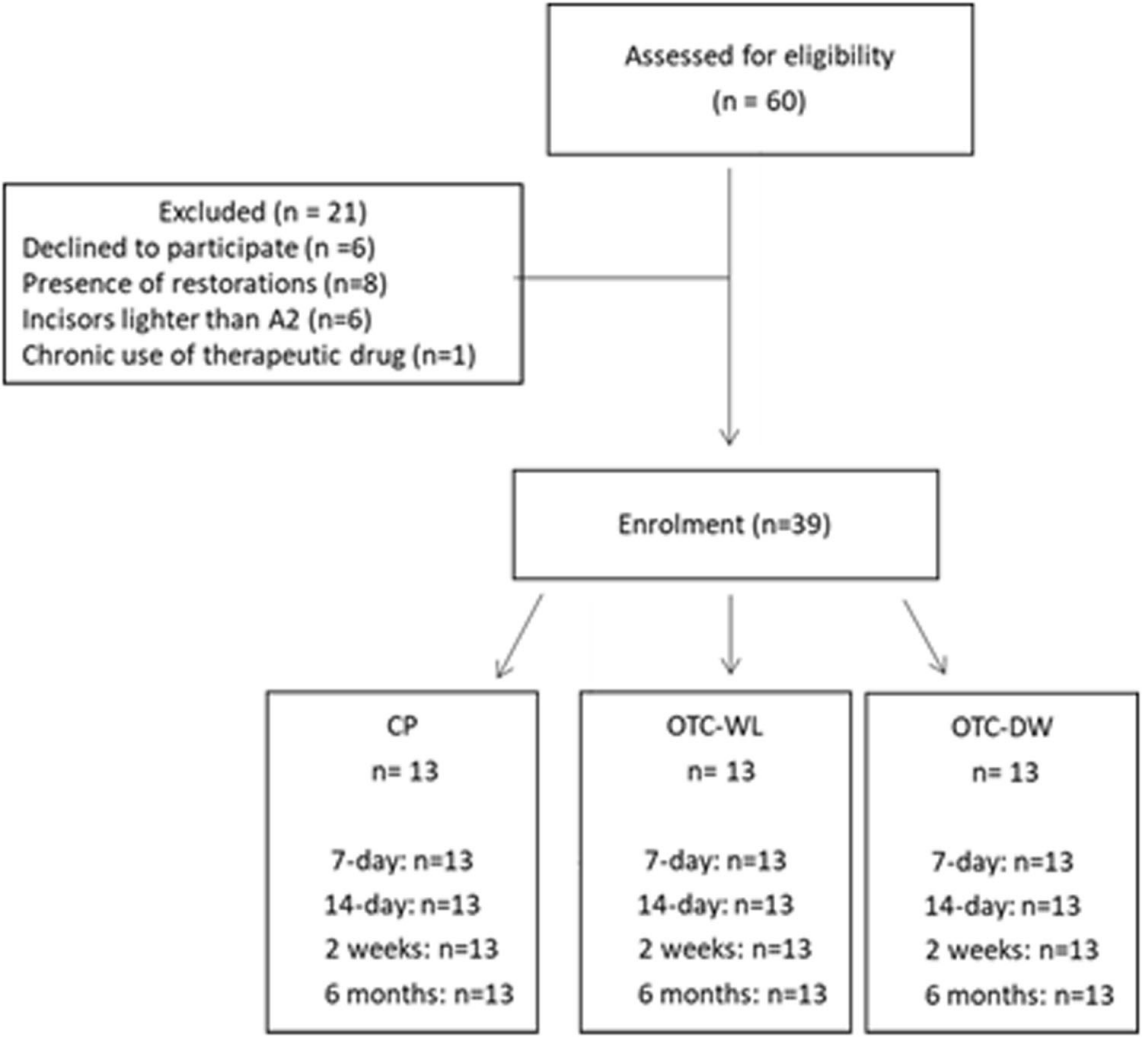
CONSORT flow diagram of the experiment.

A total of 60 volunteers were screened for eligibility. Thirty-nine of them were recruited in this trial, with an age range between 19 and 30 years (23.6 ± 3.3 mean ± standard deviation SD). Thirty-two were females (Fig. 1). The means ± standard error (SE) of the ΔE values in the three experimental groups are shown in the Table 3. There were statistically significant differences (*p* < 0.05) in ΔE values between conventional at-home whitening *vs*. OTC-DW groups at all measurement points and between conventional at-home whitening *vs*. OTC-WL at T2, T3 and T4 measurement points. No significant differences in ΔE values were detected between OTC-DW *vs*. OTC-WL at any measurement point (*p* > 0.05). The mean ± SE of the ΔE values were (9.54 ± 3.86) for the conventional at-home whitening, (4.24 ± 2.97) for OTC-WL and (2.74 ± 2.70) for OTC-DW at 14-days of whitening.

**Table 3:** ΔE means (SE) at different time points within each group.

Group	T1	T2	T3	T4
	∆E (SE)	∆E (SE)	∆E (SE)	∆E (SE)
CP	5.64 (2.42)^a^	9.54 (3.86)^a^	9.69 (3.58)^a^	9.95 (3.83)^a^
OTC-WL	3.78 (3.82)^ab^	4.24 (2.97)^b^	3.04 (2.96)^b^	2.42 (2.67)^b^
OTC- DW	1.95 (2.34)^b^	2.74 (2.70)^b^	1.79 (2.04)^b^	1.52 (2.03)^b^
*p* value	0.000	0.000	0.000	0.000

*T1* 7-day, *T2* 14-day of bleaching, *T3* 2 weeks and *T4* 6 months after bleaching.

Differences between the groups were evaluated using one-way ANOVA.

Different superscript letters (a, b) within the same column indicate the statistical significance *p* < 0.05.

Table 4 presents the means ± SE of tooth sensitivity (TS) values reported by participants according VAS scale. Significantly higher TS values were shown in conventional at-home whitening group at all measurement points (*p* < 0.05); 1-day (0.69 ± 1.03), 2-day (0.92 ± 1.26), 3-day (1.08 ± 0.86), 4-day (1.34 ± 0.96), 5-day (1.46 ± 1.39), 7-day (1 ± 1) and 14-day (2.15 ± 1.91).

**Table 4:** TS mean ± SE at different time points.

Measurement point	Group	mean ± SE	*p* value
Day-1	CP	0.69 ± 1.03	0.015
	OTC-WL	0	
	OTC- DW	0.08 ± 0.28	
Day-2	CP	0.92 ± 1.26	0.001
	OTC-WL	0.08 ± 0.28	
	OTC- DW	0	
Day-3	CP	1.08 ± 0.86	0.000
	OTC-WL	0.08 ± 0.08	
	OTC- DW	0	
Day-7	CP	1 ± 1	0.001
	OTC-WL	0.15 ± 0.38	
	OTC- DW	0	
Day-14	CP	2.15 ± 1.91	0.000
	OTC-WL	0	
	OTC- DW	0	

Differences between the groups were evaluated using Kruskal-Wallis analysis of the data.

## Discussion

The null hypothesis investigated in this trial was rejected as the results demonstrated statistical differences in efficiency and TS observed among the tested whitening systems. Conventional at-home whitening revealed significantly improved colour change when compared to the other OTC systems, in agreement with previous investigations [14, 19–21]. The higher concentration of CP (20%) and the extended application time (4 h daily) may explain this superiority. OTC-ready to use trays produced a colour change similar to that obtained using at-home whitening with CP under laboratory conditions [22]. The effect of OTC tray adaptation, the application protocol, food colour, beverages and saliva can explain the diverse outcomes reported in previous laboratory studies.

In this trial, the colour change values in OTC-paint on gel group did not exceed 2.74 at any measurement point implying that it is clinically difficult to detect any whitening effect. Human eye cannot see ∆E values below 1, and it barely distinguishes ∆E values between 2 and 3. However, ∆E values below 3.3 were suggested to be clinically insignificant [21]. Previous in-vitro and in-vivo studies showed that the colour change using OTC paint-on gel products caused a colour difference below the clinically detectable threshold [21, 23]. This might be attributed to the design of OTC-paint on gel products where there is no physical barrier to protect the active materials from oral environment and enhance the contact with the tooth surface [24]. In addition, the reduced CP concentration in these products decreases the whitening efficiency [25]. This may encourage patients to overuse these products to get the expected aesthetic outcome.

Colour relapse in the OTC-paint on gel and OTC-ready to use tray groups was detected at 2 weeks and 6 months measurement points. The reduced efficiency of OTC products may encourage patients to overuse these products to get the expected aesthetic outcomes which, in turn, causes morphological and chemical alterations in dental hard tissues [26] It has been shown that whitening products with reduced pH caused more enamel erosion and mineral loss [26]. The acidic pH values of OTC products increase enamel surface roughness, which could cause an increased extrinsic stain accumulation that led to faster shade relapse [19, 27]. In contrast, conventional at-home whitening revealed colour stability after 6 months of whitening, in accordance with previous investigations [28].

Participants in the at-home whitening group reported higher TS when compared to OTC-paint on gel and OTC-ready to use trays groups. This finding has been observed in a previous study that compared at-home bleaching with OTC strips and pre-filled trays [29]. The peroxide penetration into the pulp in conjunction with the increase of enamel/dentine permeability may initiate TS [27]. The CP application period in at-home bleaching was considerably longer than that in OTC groups. The CP concentration in both tested OTC products was not declared by manufacturers. Thus, it can be hypothesised that the low efficiency and reduced TS in OTC groups may be linked to sub-optimal concentration of active agent [20].

## Conclusion

Conventional at-home whitening revealed significantly improved shade change when compared to the OTC-paint on gel and OTC-ready to use tray whitening systems. The shade change in OTC-paint on gel group did not exceed the clinically detectable threshold at any measurement point. Participants in OTC whitening groups reported less TS. There was a significant colour relapse in OTC groups after 2 weeks and 6 months of whitening. Conventional at-home whitening using 20% CP showed higher colour stability.

## Data Availability

The data that support the findings of this study are available on request from the corresponding author.
